# Directing Cholangiocyte Morphogenesis in Natural Biomaterial Scaffolds

**DOI:** 10.1002/advs.202102698

**Published:** 2021-11-16

**Authors:** Quinton Smith, Jennifer Bays, Linqing Li, Haniyah Shareef, Christopher S. Chen, Sangeeta N. Bhatia

**Affiliations:** ^1^ Howard Hughes Medical Institute Chevy Chase MD 20815 USA; ^2^ David H. Koch Institute for Integrative Cancer Research Massachusetts Institute of Technology Cambridge MA 02142 USA; ^3^ Department of Bioengineering Boston University Boston MA 02215 USA; ^4^ The Wyss Institute for Biologically Inspired Engineering Harvard University Boston MA 02215 USA; ^5^ Institute for Medical Engineering and Science Massachusetts Institute of Technology Cambridge MA 02142 USA; ^6^ Department of Electrical Engineering and Computer Science Massachusetts Institute of Technology Cambridge MA 02142 USA

**Keywords:** biomaterials, cholangiocyte morphogenesis, liver, microfluidics, morphogenesis

## Abstract

Patients with Alagille syndrome carry monogenic mutations in the Notch signaling pathway and face complications such as jaundice and cholestasis. Given the presence of intrahepatic ductopenia in these patients, Notch2 receptor signaling is implicated in driving normal biliary development and downstream branching morphogenesis. As a result, in vitro model systems of liver epithelium are needed to further mechanistic insight of biliary tissue assembly. Here, primary human intrahepatic cholangiocytes as a candidate population for such a platform are systematically evaluated, and conditions that direct their branching morphogenesis are described. It is found that extracellular matrix presentation, coupled with mitogen stimulation, promotes biliary branching in a Notch‐dependent manner. These results demonstrate the utility of using 3D scaffolds for mechanistic investigation of cholangiocyte branching and provide a gateway to integrate biliary architecture in additional in vitro models of liver tissue.

## Graphical abstract

The utility of natural polymer blends, coupled with developmentally relevant chemical cues, is appraised for their ability to direct branching morphogenesis in primary human cholangiocytes. The resulting structures from crosstalk between EGF and Notch signaling, can be ablated using CRISPR‐Cas9 editing, and organized within a 3D microfluidic device.

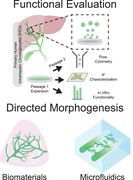

## 1 Introduction

The liver is the largest internal organ in the body and is responsible for performing over 500 different vital functions. These tasks include detoxifying drugs, storing nutrients, and producing essential factors such as albumin, clotting proteins, and bile. At a microscopic view, the liver organization consists of repeated hexagonal units termed hepatic lobules. These lobules contain sheets of hepatocytes, flanked by six portal triads, which are comprised of a network of portal veins, hepatic arteries, and intrahepatic bile ducts. In this triad, the vasculature is responsible for the transport of oxygen, nutrients, and clotting factors. The bile ducts, on the other hand, transport hepatocyte‐secreted bile acid to the small intestine. Efforts to study liver biology benefit from in vitro model systems that recapitulate aspects of the tissue's native cellular composition and architecture. Existing liver tissue engineering strategies have successfully incorporated human vascular networks with primary hepatocytes in vitro.^[^ ^1^, ^2^, ^3^, ^4^ ^]^ However, cell sourcing remains a critical bottleneck in our ability to study human intrahepatic biliary biology. Coupled with this limitation, in vitro model systems of the biliary system primarily rely on rodent isolates,^[^ ^5^ ^]^ immortalized cell lines,^[^ ^6^, ^7^ ^]^ or adult/pluripotent stem cell‐derivatives.^[^ ^8^, ^9^, ^10^, ^11^, ^12^, ^13^, ^14^ ^]^ These populations either do not display mature cholangiocyte marker expression or are limited to forming a non‐perfusable structure that lacks the branched architectures found in the native liver.

Immortalized mouse hepatoblasts, with the capacity to differentiate into hepatocytes or cholangiocytes, have been shown to form cystic ductal structures in extracellular matrix (ECM) conditions that contain both laminin‐rich Matrigel and rat tail collagen I. This finding contrasts with other epithelia, such as Madin‐Darby canine kidney cells (MDCK), which only require integrin engagement with collagen I motifs to polarize and expand as cysts.^[^ ^15^ ^]^ Notably, when mouse hepatoblasts are cultured in collagen I, they form branch‐like structures but are unable to polarize. Cystic efficiency in hepatoblast culture is dependent on EGF and hepatocyte growth factor (HGF) stimulation, as well as metalloproteinase and TGF*β* activity.^[^ ^16^ ^]^ Consistent with these findings, immortalized progenitor‐like small cholangiocytes derived from mice cannot spread in collagen I matrices or form cystic structures in Matrigel but require decellularized liver ECM to undergo branching morphogenesis.^[^ ^17^ ^]^ To reduce the complexity of xeno‐derived matrices, synthetic hydrogel scaffolds can be engineered with specific material properties such as stiffness, porosity, and adhesion densities, permitting systematic decoupling of physicochemical cues necessary for tissue homeostasis and morphogenesis. For example, immortalized normal rat intrahepatic cholangiocytes (NRCs)^[^ ^7^ ^]^ encapsulated in polyethylene glycol (PEG) pre‐polymers that are functionalized with fibronectin‐derived RGD binding motifs can expand as cysts in a stiffness‐dependent manner. Soft (0.5 kPa) hydrogel matrices lead to frequent cyst formation, and increased RGD concentrations encourage multi‐lumenal features, but interconnected branched epithelial structures could not be generated.^[^ ^18^ ^]^ Furthermore, a variety of approaches have been used to build perfusable biliary tubes, but the resulting channels lack hierarchical structure, and are composed of rodent‐derived cholangiocytes.^[^ ^19^, ^20^ ^]^ To date, the vast majority of studies in this field have relied on rodent‐derived cellular material, and there are many examples in the liver tissue engineering field in which findings obtained using mouse and rat cells do not correlate with the outcomes obtained with human samples.^[^ ^21^, ^22^, ^23^, ^24^, ^25^ ^]^ While the advent of immortalized human biliary cell lines can help to reduce these variances, they present their own limitations, including a heavy mutational burden that can lead to clonal variability from the original source and transformation from the natural phenotype as in the case with hepatocytes.^[^ ^26^ ^]^ Motivated both by these advances and the remaining progress gaps, we sought to build upon existing biliary platforms and investigated the potential to fabricate branched human cholangiocyte networks, alongside cholangiocyte‐lined channels.

To this end, we characterized adult‐derived primary human cholangiocytes and investigated their branching potential in 3D culture conditions. First, we performed cellular profiling with biliary‐specific surface markers and measured tissue‐specific enzymatic activity. After validating cholangiocyte‐like identity of these isolates, we performed 3D culture experiments in natural biomaterial scaffolds containing varying concentrations of mitogens relevant to liver development and regeneration. From these studies, we found that growth factor cues and the extracellular matrix coax in vitro biliary network assembly in a Notch signaling‐dependent manner. Next, to build physiologically relevant biliary networks with hierarchical architecture, we looked to our previously established organ‐on‐chip model systems.^[^ ^1^, ^27^, ^28^ ^]^ We were able to create on‐demand 300 µm ductal structures by casting extracellular matrix around needle templates and removing the needles post polymerization. When configured to permit bulk biliary network assembly, we were able to manufacture both large and small vessel‐like architectures. Collectively, our combined approaches reinforce the role of EGF stimulation and Notch signaling in biliary morphogenesis. While Notch signaling has been thoroughly investigated in its role in directing intrahepatic biliary branching during development, there is limited evidence of translating these findings into a human relevant in vitro system. Here, we emphasize a materials and cellular characterization approach, demonstrating the potential to investigate additional aspects of cholangiocyte functionality including permeability, shear stress response, and transport of bile fluid components such as cholic/chenodeoxycholic acids or xenobiotics.

## 2 Results

### 2.1 Primary Intrahepatic Cholangiocytes Maintain Functional Marker Expression In Vitro

**Figure 1:**
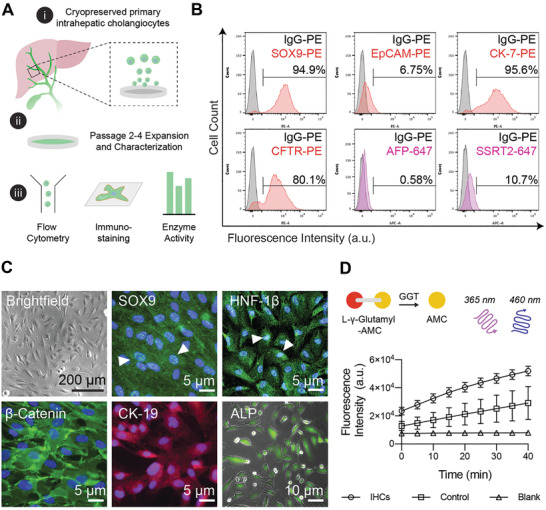
Characterization of primary intrahepatic cholangiocytes. A) i) Schematic describing the approach to characterize adult, cryopreserved primary human intrahepatic cholangiocyte (IHCs); ii) IHCs were expanded on collagen‐coated plates and used for up to four passages; iii) Passage 2 IHCs were characterized with a combination of flow cytometry analysis, immunofluorescence staining, and enzymatic activity assays. B) Representative flow cytometry histogram plots showing SOX9, EpCAM, CK‐7, CFTR, AFP, and SSRT2 expression compared to IgG isotype controls (in gray; [*n* = 3]). C) Representative brightfield and epi‐fluorescence images of IHCs cultured on collagen‐coated substrates (*n* = 3 biological replicates). IHCs show nuclear localization of SOX9 and HNF‐1*β* expression (green signal overlapping with nuclear DAPI stain in blue; white arrows), and positive cytoplasmic expression of *β*‐catenin (green) and CK‐19 (red). IHCs were incubated with a live alkaline phosphatase (ALP) stain for 30 min, washed with serum‐free media and imaged with fluorescence microscopy (nuclei were stained with DAPI). D) *γ*‐glutamyl transferase activity (GGT) measured using a colorimetric assay where time course of fluorescence intensities is shown, denoting the liberation of 7‐amino‐4‐methyl coumarin (AMC) from a *γ*‐glutamyl quenched substrate. Graphs represent mean and standard deviation from technical triplicates with blank and GGT positive controls.

Sourcing human primary cholangiocytes remains a challenge owing to their intrahepatic localization, however pluripotent stem cell derivatives^[^ ^10^, ^29^, ^30^, ^31^ ^]^ or Lgr5+ enriched adult duct progenitor populations acquired from biopsied samples,^[^ ^9^ ^]^ have been routinely used as model systems to date. While these sources permit the expansion of cholangiocyte‐like cells as cystic organoids, their maintenance requires administration of a complex chemical milieu that maintains a stem‐like state and does not promote the self‐assembly of physiologically relevant branched architectures. Here, we appraised morphological and functional features of commercially available, adult intrahepatic biliary epithelial cells (IHCs) using a combination of tools including gene expression analysis, flow cytometry, and immunofluorescence staining (**Figure** **1**A). Phenotypic assessment was performed on thawed biliary cells that were expanded on collagen type I coated plates for up to four passages. We first performed flow cytometry analysis to assess the homogeneity of these cell populations, as well as the degree of mature protein marker expression. As expected, the majority of IHCs expressed biliary markers SRY‐related HMG transcription factor 9 (SOX9), cytokeratin 7 (CK‐7), cystic fibrosis transmembrane conductance regulator (CFTR), and a subset were positive for epithelial cell adhesion molecule expression (EpCAM). In contrast with stem cell‐derived sources, IHCs expressed low levels of the mature marker somatostatin receptor 2 (SSRT‐2), a mediator of hormonal signals during digestion, and did not exhibit alpha‐fetoprotein (AFP), a common marker expressed by progenitors (Figure 1B).

We observed that cultured IHCs exhibited polygonal morphology, with visible cell–cell adhesions (Figure 1C). While IHCs initially grew as distinct patches with regular borders, after multiple passages they began to exhibit elongated and spindle‐like morphologies, indicative of epithelial to mesenchymal transition, and acquired some fibroblastic features such as fibronectin deposition while maintaining a degree of junctional marker expression (Figure S1, Supporting Information). Based on this observation, IHCs were not used for functional studies beyond five passages. Immunofluorescence microscopy was used to visualize the localization and expression of proteins common to epithelial and cholangiocyte identity, namely membrane bound cell–cell adhesion marker *β*‐catenin, cytoplasmic cytokeratin 19 (CK‐19), and nuclear biliary markers hepatic nuclear factor‐1*β* (HNF‐1*β*) and SOX9 (Figure 1C). Zinc metalloenzymes alkaline phosphatase (ALP) and *γ*‐glutamyl transpeptidase (GGT) are present in nearly all tissues but are enriched in biliary epithelium in vivo and we observed that this population of IHCs demonstrate these functional features (Figure 1C,D; Figure S1, Supporting Information). Collectively, through analysis of protein expression and enzymatic activity, we presume that this biliary population consists mainly of large cholangiocytes compared to a more plastic small cholangiocyte population. This interpretation is based on the observations that the populations analyzed express CFTR, and exhibit GGT and ALP activity, which is enriched in large cholangiocytes.

### 2.2 Composite Extracellular Matrices Promote Cholangiocyte Branching in 3D Culture

**Figure 2:**
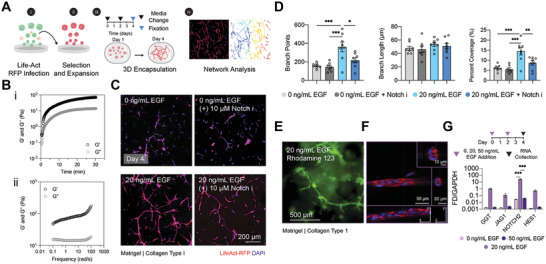
Composite Matrigel and collagen type 1 gels support cholangiocyte branching morphogenesis. A) Experimental schematic to probe the capacity for primary human intrahepatic cholangiocytes to undergo branching morphogenesis: i) Fluorescence tagging of IHCs by rLVUbi‐LifeAct‐TagRFP cytoskeletal labeling; ii) a homogenous population of IHCs stable for red fluorescence protein (RFP) expression of F‐actin was generated after puromycin selection and expanded in cholangiocyte media; iii) resulting populations were encapsulated in Matrigel/collagen type 1 gels for 4 d; and iv) features of branching morphologies were measured from maximum intensity z‐projections of confocal images using a segmentation algorithm. B) Network formation kinetics and viscoelastic behavior of Matrigel/collagen type 1 composite gels. i) Viscoelastic modulus of composite gels (*G*′, storage modulus and *G*″, loss modulus) measured with a dynamic time sweep. ii) Dynamic frequency sweep of composite gels. C) Representative images of LifeAct RFP‐IHCs encapsulated in Matrigel/Collagen I blends, cultured with and without EGF or Notch inhibition (10 × 10^−6^ m L,685458). D) Individual data points of quantified branch length, points, and network percent coverage. *P*‐values were obtained using one‐way ANOVA Tukey's hypothesis testing. Representative images generated from at least seven independent fields of view from three biological replicate experiments. At least 20 segmented features were analyzed per field of view. E) Functional uptake of Rhodamine 123 after 4 d of IHC culture in Matrigel/collagen type 1 blends containing 20 ng mL^−1^ of EGF. F) F‐actin stain of cholangiocytes grown in Matrigel/collagen type 1 composites after 4 d with 20 ng mL^−1^ EGF stimulation. G) mRNA expression levels of GGT and Notch signaling genes measured via RT‐qPCR for IHCs 4 d post 3D culture. Bar graphs show internal triplicate measurements from the pooled collection of five biological replicate gels. *P*‐values were obtained using two‐way ANOVA Tukey's hypothesis testing. *P* < 0.033 (*), *P* < 0.002 (**), *P* < 0.001 (***). All data are represented as mean ± SEM.

After validating that IHCs exhibit a collective set of phenotypic and functional traits, we proceeded to leverage insight from in vivo developmental studies to assay whether these cholangiocyte‐like cells can self‐assemble into biliary networks in vitro. We hypothesized that IHCs would have the ability to form interconnected 3D branched network structures within a native extracellular niche, given the appropriate introduction of matrix and chemical cues. To this end, we first fluorescently labeled IHCs using a puromycin‐selective lentiviral red fluorescent protein (RFP) system, designed to visualize live F‐actin expression. Next, after selection via antibiotic resistance, we expanded and encapsulated the resulting Life‐Act RFP‐IHCs at a density of 1 × 10^6^ cells mL^−1^ in either Matrigel or Matrigel/collagen type 1 blends. Matrigel is a complex laminin‐rich extracellular matrix composite derived from mouse sarcoma cells and is a well‐known biomaterial that supports epithelial tissue polarization. Given its complexity, Matrigel mimics the basement membrane extract, which separates epithelium from the interstitium. We hypothesized that composite gels, consisting of 50% (v/v) Matrigel and 3 mg mL^−1^ of collagen type 1, would promote branching morphogenesis via the fibrillar architecture of collagen matrices.^[^ ^32^ ^]^ Four days (d) post encapsulation, we fixed and labeled the nuclei of resulting structures, imaged using confocal microscopy, and performed image analysis on z‐plane maximum intensity projections. Samples cultured for longer than 4 d collapsed owing to collagen condensation, as demonstrated in other self‐assembled structures.^[^ ^33^ ^]^ To evaluate the resulting morphological features, we used a computer algorithm to segment image attributes and quantified network coverage, branch points, and structure lengths (**Figure** **2**A).

IHCs that were encapsulated in growth factor‐reduced Matrigel were incubated with or without EGF stimulation and exposed to conditions with L‐685458, a potent and selective *γ*‐secretase inhibitor that blocks Notch transcriptional activity.^[^ ^34^ ^]^ IHCs cultured in 100% Matrigel scaffolds without EGF stimulation produced limited sprouting and formed large aggregate structures, with multiple branched features extending from the clustered core. With Notch inhibition, cell aggregation diminished, indicative of increased matrix interaction compared to homotypic cell‐cell interactions. However, multicellular branched features were not identified (Figure S2, Supporting Information). Sprouting behavior in Matrigel improved in the presence of 20 ng mL^−1^ EGF, demonstrating the role of mitogen stimulation on biliary branching in laminin‐rich matrices (Figure S2, Supporting Information).

We hypothesized that the addition of fibrillar architecture within Matrigel could promote biliary sprouting behavior. To evaluate the viscoelastic properties of the composite hydrogels consisting of Matrigel and 3.0 mg mL^−1^ rat tail collagen type I, we performed dynamic time and frequency sweeps using shear rheology (Figure 2B). From these measurements we found that growth factor‐reduced Matrigel had a Young's modulus of 29 ± 6 Pa, while hydrogel blends had a Young's modulus of 75 ± 7 Pa (Figure S3, Supporting Information). Following biomaterial characterization, we assessed IHC sprouting behavior under EGF stimulation and Notch inhibition (Figure 2C). While there were no significant differences in the number of branch points, branch length, or percent coverage of IHCs cultured in 3D composite gels with 0 ng mL^−1^ EGF with or without Notch inhibition, we found that the addition of 20 ng mL^−1^ EGF had a significant impact on the potential to form interconnected branched features. Notably, we observed an increase in the density and number of observed branched points (Figure 2D). We also evaluated the combinatorial role of co‐administering HGF with EGF on IHC branching potential, as they both have been implicated in inducing ductal morphogenesis during development (Figure S4A, Supporting Information). We found that HGF alone can also support IHC sprouting, but the combination with HGF and EGF leads to densely interconnected structures. Again, when Notch inhibition is introduced, the additive effects of HGF and EGF are abrogated, implicating a strong role for Notch signaling in branching morphogenesis (Figure S4B,C, Supporting Information).

We next appraised network functionality through analysis of ATP‐dependent flux. The multidrug resistance 1 (MDR1) P‐glycoprotein protects cholangiocytes from toxic cationic agents present in hepatocyte‐secreted bile acid, including xenobiotic substances or drugs. Rhodamine 123 is a fluorescent tracer dye and substrate of MDR1 and is actively transported into the lumen of biliary epithelial cells. 3D incubation of Rhodamine 123 in 20 ng mL^−1^ EGF‐stimulated networks led to secretory functionality, with a lumenal influx of the fluorescent substrate (Figure 2E). We were also able to identify lumens within the branching cholangiocytes using high‐resolution confocal microscopy (Figure 2F). For a controlled evaluation of the role of growth factor presentation during IHC branching, we chose to specifically look at EGF stimulation in 3D culture conditions, but also compared these effects to IHCs cultured as 2D monolayers (Figure S5, Supporting Information). To elucidate the effects of EGF on known Notch signaling targets, IHC transcript levels were measured in 3D composite gels cultured with 0, 20, or 50 ng mL^−1^ EGF. Our analysis revealed that EGF stimulation affected GGT, JAG1, NOTCH2, and HES1 mRNA expression levels in a dose‐dependent manner and elicited a significant increase in NOTCH2 gene expression with 20 ng mL^−1^ EGF stimulation (Figure 2G).

### 2.3 EGF Stimulation Enhances Notch Signaling during Cholangiocyte Branching

**Figure 3:**
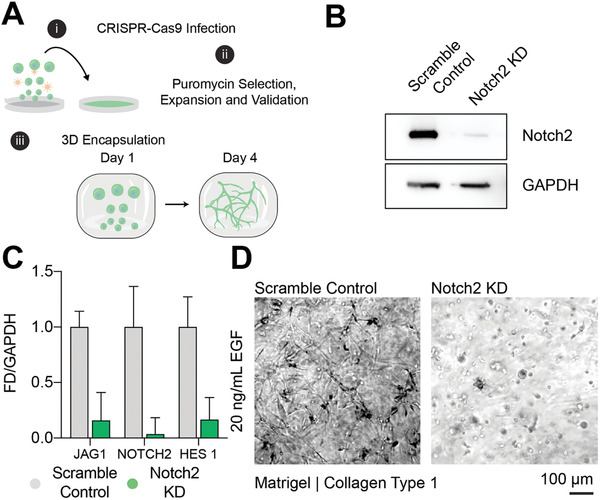
CRISPR‐Cas9 mediated Notch2 knockdown ablates cholangiocyte branching morphogenesis in hydrogel blends. A) Schematic of the workflow to test the impact of Notch2 knockdown in cholangiocyte branching morphogenesis. B) Notch2 intracellular domain (Notch2 – ICD) downregulated protein expression in Notch2 knockdown (KD) IHCs compared to scramble control cells. C) Comparison of mRNA expression between scramble control and Notch2 KD cells. Notch2 KD cells show downregulation of JAG1, NOTCH2, and HES1 gene expression compared to scramble controls (*n* = 2). D) Brightfield images of scramble control and Notch2 KD cells grown in Matrigel/collagen I hydrogel blends after 4 d. Notch2 KD cholangiocytes show blunted branching compared to control cells (*n* = 3, triplicate gels).

We engineered Notch2‐deficient cells using CRISPR/Cas9 (clustered regularly interspaced short‐palindromic repeats/CRISPR associated protein 9) mediated deletion to further validate EGF's role in Notch signal transduction and IHC branching potential. In brief, two guide RNAs (gRNAs; Scramble control, and Notch2) were cloned into puromycin‐sensitive lentiviral CRISPR/Cas9 vectors and packaged with HEK293 cells. The resulting complexed particles were used to infect freshly thawed IHCs (**Figure** **3**A). Relative protein levels of the Notch2 intracellular domain (Notch2‐ICD) were confirmed via Western blot analysis of cell lysates, showing decreased expression in Notch2 knockdown (KD) compared to scrambled control cells (Figure 3B). These changes were consistent at the mRNA level (Figure 3C), reflecting a broad uptake of the CRISPR‐mediated deletion, despite some residual, Notch2‐intact cells remaining in the population. Finally, cells from the control and Notch2 depleted populations were encapsulated in Matrigel/collagen type 1 composite gels with 20 ng mL^−1^ EGF stimulation. After 4 d, the Notch2‐depleted population exhibited dramatically blunted sprouting potential, relative to the scrambled control cells (Figure 3D). These results confirm the role of EGF in the Notch signaling axis in IHCs, which presumably mediates branching morphogenesis in composite natural scaffolds.

### 2.4 Intrahepatic Biliary Tree on a Microfluidic Chip

**Figure 4:**
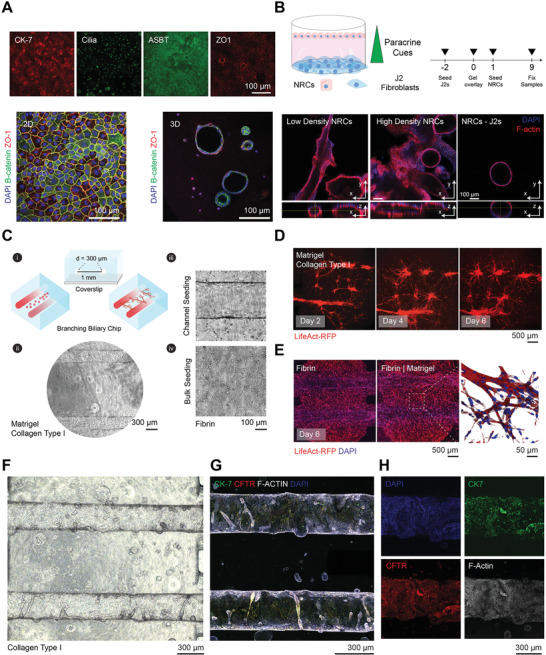
Intrahepatic biliary ducts on a chip. A) Immunofluorescence images, characterizing marker expression and morphology of normal rat cholangiocytes grown on planar 2D surfaces and in 3D type 1 collagen gels (3 mg mL^−1^). B) Experimental schematic describing the co‐culture of J2 fibroblasts and NRCs separated by a collagen gel. In addition, representative confocal images of NRCs after 9 d of culture are shown (with NRCs cultured at low density [20 000 cells per well] and high density [50 000 cells per well]), highlighting the ability to form branched lumenized structures with stromal support (*n* = 3 gels). C) i) Device schematic and representative brightfield images, showing top and cross‐sectional views of a dual channel microfluidic platform; ii) encased within the biomaterial scaffolds are two parallel 300 µm open lumenal structures spaced 1 mm apart; iii) the arrangement provides capacity to seed IHCs and flow media in the patent channels; and iv) encapsulate IHCs in the bulk compartment of the device. D) Representative time course images of LifeAct RFP‐IHCs grown in microfluidic device with 20 ng mL^−1^ EGF, showing collapse of open structures after 6 d, but anastomosis between bulk networks with channel structures. E) Representative maximum intensity projection images of LifeAct RFP‐IHCs cultured for 6 d with 20 ng mL^−1^ EGF in fibrin scaffolds, showing maintenance of open cell‐laden channels compared to Matrigel/collagen type 1 scaffolds (nuclei stained with DAPI). F) Representative brightfield image of microfluidic cultured NRCs (*n* = 3 devices). G) Confocal z‐projection alongside H) an inset of microfluidic cultured NRCs showing polarized epithelium along the channel walls (*n* = 3 devices).

By using a reductionist approach to investigate the contribution of matrix mechanics, composition, and growth factor stimulation, we were able to develop a framework to describe the factors that promote in vitro IHC branching phenotypes. The outcome of these networks, however, do not fully recapitulate a strong polarized phenotype, nor mirror developmental morphogenesis, which results from the crosstalk between epithelial and stromal populations. While hepatoblast specification to cholangiocytes relies on Notch signaling, paracrine cues from the vascular and stromal populations of the portal vein help to direct hepatic fate and subsequent ductal lumenogenesis in vivo. We investigated the potential to recapitulate some of these developmental processes, leveraging normal rat cholangiocytes (NRCs), which represent a strongly polarized, yet immortalized intrahepatic biliary population. When NRCs were cultured in 3D matrices composed of collagen type 1, they were able to form polarized cystic structures (**Figure** **4**A). However, matrix composition alone, or coupled with growth factor supplementation, could not induce branching features as in the case of IHCs (data not shown). However, when NRCs were cultured in the presence of stromal cells, they were able to form large lumenized branched structures. While promising, both approaches which contain either cystic structures or uncontrolled lumenogenesis, lack architectural control and intralumenal accessibility (Figure 4B). To this end, we used a previously described polydimethylsiloxane (PDMS)‐based microfluidic platform, to build intrahepatic biliary systems, that allows simultaneous control of large ductal architecture and intralumenal flow.^[^ ^27^, ^35^ ^]^

In brief, this PDMS‐based device contains guide features when bonded on a coverslip, which permits the insertion of parallel needles that are spaced 1 mm apart, with a diameter of 300 µm. Upon device assembly, the needles are inserted into the microfluidic chamber where prepolymer solutions with or without cells can be introduced. Following polymer crosslinking, the needles can be removed leaving open structures that can be seeded with cells, allowing for bulk cellular self‐assembly and prefabricated vessel‐shaped structures (Figure 4C). We first leveraged insight from our bulk 3D culture experiments, and mixed IHCs in pre‐polymerized composite gels into the device. Following polymerization and needle removal, we seeded additional IHCs into the lumen of the channels via a pressure differential and cultured the devices under gravity‐driven perfusion using a rocker platform. After monitoring RFP expression over the course of 6 d, we found that the vessel structures collapsed into cords, blunting channel access for perfusion, but permitted the assembly of branching architectures in the bulk of the device (Figure 4D). When comparing this observed phenotype to NRCs cultured within the microfluidic device, we found that the matrices supported bulk cystic morphogenesis and sustained polarized cell‐laden channels as previously described (Figure 4F–H; Movie S1, Supporting Information).^[^ ^19^ ^]^ To maintain patent vessel architecture, we explored the use of alternative biomaterial scaffolds. We found that fibrin gels, a sticky clotting agent comprised of fibrinogen and thrombin, enhanced biomaterial adhesion to the glass and PDMS features of the device, sustaining both patent cholangiocyte‐lined ducts as well as dense, interconnected branched features (Figure 4E). Collectively, these results demonstrate the capacity to use microfluidics as a tool to control both architecture and perfusion of epithelial structures.

## 3 Discussion

Here, we describe a pipeline for characterizing primary human biliary isolates and appraised their functional and morphogenic potential under controlled in vitro microenvironments. We specifically show that extracellular matrix composition, growth factor presentation, and Notch activity elicit the self‐assembly of branched epithelial structures that mimic native biliary tissue. The resulting structures maintain cholangiocyte‐specific function and can transport multiple drug resistance protein substrates. Importantly, we can combine top‐down and bottom‐up approaches to engineer both large (300 µm) and smaller self‐assembled branched cholangiocyte networks in a microfluidic platform that can be leveraged for future evaluation of shear stress, permeability, and spatially organized co‐cultures. Collectively, these results reinforce approaches that can be used to study cell–cell and cell–matrix during tissue morphogenesis and provides a strategic framework for future integration of biliary epithelium in existing engineered liver platforms.^[^ ^36^, ^37^, ^38^ ^]^

Notch signaling is an evolutionarily conserved intracellular pathway that regulates many aspects of embryonic development including cell‐fate specification and morphogenesis. Consequently, dysregulation of Notch signaling can lead to an array of developmental disorders that affect many tissues including the liver. Mammalian cells contain four different single pass transmembrane Notch receptors (NOTCH 1–4). Upon ligand engagement by neighboring Jagged or Delta‐like protein expressing cells, the extracellular domain of the Notch receptor undergoes proteolytic cleavage by an ADAM metalloprotease. Following this reaction, cleavage of the Notch intracellular domain (NICD) by *γ*‐secretase, liberates the protein to translocate to the nucleus where it interacts with the recombination signal binding protein for immunoglobulin kappa J (RBPJ) transcription factor. This association promotes downstream expression of hairy and enhancer of split‐1 (HES1) which acts to confer instructions to neighboring cells during embryonic patterning. Notch signaling is essential to bile duct specification and subsequent tubule formation,^[^ ^39^ ^]^ and patients with Alagille syndrome, contain inherited mutations in either the Notch2 receptor or Jag1 ligand resulting in cholestasis from ductal paucity.^[^ ^40^ ^]^ Kitade et al. show mouse derived bipotential hepatic progenitor cells (HPCs) undergo cholangiocyte specification and branching morphogenesis in Matrigel/collagen I gels. This branching is mediated by EGFR and MET stimulation using EGF and HGF media supplementation, respectively. EGFR null HPCs are unable to undergo branching morphogenesis or acquire biliary marker expression upon growth factor addition.^[^ ^41^ ^]^ Furthermore, EGFR competent HPCs with Notch deletion fail to differentiate or branch with MET/EGFR activation. In line with these results, our human in vitro system corroborates the crosstalk between Notch/EGF (EGFR) signal transduction in biliary morphogenesis.^[^ ^39^, ^42^ ^]^ In the system described here, we find that Matrigel, a composite ECM that is enriched with basement membrane extract, is unable to induce IHC polarization or branching morphogenesis. This differential response can be attributed to an array of factors, including the primary cell source used in this study. Another plausible rationale for the observed behavior of IHCs in this system can be attributed to liver matrix biology. The liver does contain ECM components including collagen, fibronectin, and proteoglycans; however, the liver is cell dense and contains minimal ECM presentation. In fact, the portal spaces, where the vascular tracts and bile ducts are located have little to no ECM. Furthermore, it is speculated that the ECM which is present in the biliary domains is produced by the cholangiocytes themselves.^[^ ^43^ ^]^ In vivo evidence shows that the ECM of the extrahepatic biliary system consists of heparan sulfate, proteoglycans, peanut agglutinin, laminin, collagen type IV, and nidogen.^[^ ^44^ ^]^ However, progenitors of intrahepatic biliary epithelial cells do not express these proteins. While immortalized cell lines such as NRCs serve as a viable model to study biliary biology, their distinct morphogenic potential compared to other immortalized epithelium such as MDCK cells has yet to be elucidated. By directing our efforts toward studying primary human isolates, we hope to provide motivation to build systems that allow direct comparison between animal and human biological processes.

We show that inhibition of *γ*‐secretase by L‐685458 or knockdown of Notch2 via CRISPR/Cas9 leads to reduced branching morphogenesis in vitro and abrogates transcriptional activation of HES1. Furthermore, we find that EGF stimulation during branching morphogenesis is correlated with increased Notch signal transduction, evidenced by the upregulation of HES1. We find that this transcriptional regulation of Notch activity is specific to 3D culture conditions. Whether this phenotype is regulated by ECM stiffness has yet to be elucidated, but mechanical forces have been demonstrated to regulate YAP/TAZ control of Notch activation in epidermal stem cells.^[^ ^45^ ^]^ Additionally, bi‐potent mouse hepatoblasts have been shown to preferentially differentiate into cholangiocytes at the periphery of circular micropatterned domains. Compared to central areas, these regions elicit increased actomyosin stress and elevate NOTCH2 and JAG1 transcriptional activity.^[^ ^46^ ^]^ Applying these principles to our system, we stipulate that rheological characterization of our optimized matrices will provide additional insight into the role of scaffold mechanics on Notch‐mediated morphogenesis.

Aside from standardizing media components, differences in the described morphogenic potential of varying cholangiocytes should be reconciled by transcriptional profiling, which allows for stratification of specie specific phenotypes. In addition, single cell analysis can illuminate functional differences between stem cell‐derived, adult progenitor and small/large cholangiocyte subtypes.^[^ ^47^ ^]^ Finally, while immortalized cell lines have proven useful for in vitro mechanistic studies, questions regarding their phenotypic stability, tumorgenicity and transcriptional landscape remain, limiting their clinical utility for regenerative medicine applications. Our system provides an important framework for directing primary human biliary assembly that can be integrated with existing approaches in liver tissue engineering. There are notable limitations in this system, including the need for further functional validation of the cholangiocyte‐like cells used in this study. For example, optimization of culture conditions should be conducted to limit mesenchymal features after serial passage and investigation of other markers such as the sodium‐dependent bile acid transporter (ASBT) and osteopontin (OPN) should be pursued. With this described microfluidic system, one can measure cholangiocyte permeability, response to small molecules (ATP and acetylcholine stimulation on calcium influx dynamics), shear stress, and varying bile compositions. While we demonstrate the role of EGF stimulation on Notch signaling, other signal transduction pathways such as PI3K/Akt and MEK/ERK intracellular pathways can be affected by EGF as well. Furthermore, tethering EGF ligands to the scaffolds, rather than bulk administration, could lead to enhanced signal transduction and morphogenetic processes.^[^ ^48^ ^]^ In summary, we provide insight into growth factor‐mediated branching in liver epithelium, consistent with findings in lung or kidney morphogenesis, but unveil tissue‐specific ligand‐receptor feedback between EGF and Notch signaling. Leveraging this insight, we directed the assembly of biliary duct structures using a microfluidic platform, adding a new model system of the portal triad.

## 4 Experimental Section

### Cell Culture

Human IHCs passage 1–5 (ScienCell, Carlsbad, CA) were cultured in complete epithelial growth media (ScienCell) on 50 µg mL^−1^ type I collagen coated surfaces (Corning). IHCs were isolated from human liver tissue using mechanical dissociation and enriched for CK‐19. Media was exchanged every 2–3 d, passaged at 80% confluency with 0.05% trypsin/EDTA (ThermoFisher Scientific, Waltham, MA), and maintained in a humidified 5% CO_2_ incubator at 37 °C. The primary human cells (intrahepatic cholangiocytes from ScienCell and gallbladder isolates from MGH Cell Core Resource Center) that were obtained commercially and utilized in this study were obtained from donors with signed consent, and were isolated according to established protocols. In addition, these organizations performed human tissue collection under compliance with federal, state, and local laws and regulations. Normal rat cholangiocytes were a gift from Nicholas LaRusso (Mayo Clinic) and were obtained ethically, using established isolation protocols. They were cultured as previously described.^[^ ^5^ ^]^ J2‐3T3 fibroblasts were maintained in DMEM with high glucose, 10% bovine serum albumin and were a gift from Howard Green (Harvard Medical School).^[^ ^49^ ^]^

### Flow Cytometry

Marker expression was verified through cytometry, where PE or APC‐conjugated antibodies were stained on fixed and permeabilized cells. To harvest cells for flow analysis, serum was removed prior to adding TrypLE (Invitrogen, Waltham, MA) dissociation buffer, by washing with 1× PBS. After collection, cells were fixed with 3.7% paraformaldehyde (PFA) for 5 min, spun for 2 min at 200× G, and resuspended in 0.1% Triton X for 10 min. Cells were then incubated in 100 µL of 0.1% bovine serum albumin (BSA; Sigma‐Aldrich, St. Louis, MO) in PBS with conjugated antibodies for 1 h at room temperature. Cells were washed three times to reduce nonspecific staining and analyzed on a BD LSRFortessa (BD Biosciences, Franklin Lakes, NJ). To determine levels of expression, all analyses were conducted using IgG‐PE or IgG‐APC (BD) isotype controls.

### Immunofluorescence Staining and Imaging

Cells cultured on glass collagen I coverslips were washed with 1× PBS and fixed in 3.7% paraformaldehyde for 10 min. After washing the samples with 1× PBS, samples were incubated in 0.1% Triton X‐100 (Sigma‐Aldrich) for 10 min. Next, cells were washed with 1× PBS and incubated for 1 h at room temperature in 1% BSA to block for nonspecific binding. Samples were then incubated with primary antibodies diluted in 1× overnight at 4 °C, washed with incubated secondary antibodies for 1 h at room temperature. Finally, samples were incubated with Hoechst solution (ThermoFisher) for 3 min and washed with PBS prior to imaging. Cell morphology was identified using a Nikon TE200 Inverted microscope.

### Biochemical Assays

*γ*‐Glutamyl transferase activity was acquired using the Colorimetric Assay Kit (Sigma‐Aldrich). In brief, 1 million IHCs and fibroblast controls were collected in microcentrifuge tubes. Cells were pelted by spinning down at 200× G for 3 min. After aspirating out the supernatant, cells were resuspended in 200 µL of cold GGT assay buffer and spun at 13 000× G for 10 min. Activity was acquired through kinetic absorbance measurements (418 nm) at 37 °C using a TECAN Infinite microplate reader. To determine ALP activity, growth media of live cell cultures was removed, prior to washing vessels with pre‐warmed DMEM/F‐12. Adherent cells were then incubated for 20–30 min with a 1× ALP Live Stain solution (ThermoFisher). After incubation, the ALP solution was removed, and cultures were washed two times with fresh DMEM/F‐12 for 5 min per wash prior to imaging using FITC illumination.

### Biliary Network Formation

Collagen gels were formed as previously described. In brief, 1.0 × 10^6^ cells mL^−1^ were encapsulated in gels composed of Matrigel mixed at equal parts with 3.0 mg mL^−1^ rat tail collagen type I that was titrated to pH 7.0–7.5 with 1 m NaOH. 100 µL of the collagen mixture was added to wells of a 96‐well plate and allowed to polymerize at 37 °C for 30 min. After the gel solidified, an additional 100 µL of supplemented epithelial media was added.

### Biliary Network Quantification

A custom image processing program was written in MATLAB (Natick, MA) to quantify morphological features of branching phenotypes. Maximum intensity projections of confocal z‐stacks were processed using ImageJ (NIH). Next, images were exported as JPEG files and imported into MATLAB. RFP signal was used to identify network features. Masked image segments were enumerated and evaluated for percent coverage, branch length, and branch points.

### Rheology

To determine the viscoelastic properties of the natural biomaterial scaffolds, the storage (*G*′) and loss (*G*″) moduli were determined using an AR‐G2 rheometer (TA Instruments, New Castle, DE). Acellular materials were prepared identically to cell laden samples. Frequency sweeps were conducted from 0.1 to 100 rad s^−1^. Dynamic time sweeps were conducted at a constant angular frequency of 6.27 rad s^−1^. Data were collective from at least three independent samples.

### Quantitative Reverse‐Transcription PCR

Total RNA was extracted using TRIzol Reagent (Invitrogen) from IHCs cultured under varying conditions. Quality and quantity of extracted RNA was verified by NanoDrop spectrophotometry prior to implementation of the one step RNA to Ct kit (ThermoFisher). Each measurement was conducted in triplicate with non‐template controls using a BioRad CFX96 instrument. GAPDH served as endogenous controls for global normalization to acquire mRNA expression. Reference groups for differential analysis are outlined in the text and fold differences were calculated by the comparative Ct method.

### Lentiviral‐Mediated CRISPR Genome Editing

CRISPR knockdown cells were generated using the lentiCRISPRv2 system (gift of F. Zheng, Addgene plasmid #52961). Scramble guideRNA (gRNA) (GCACTACCAGAGCTAACTCA) and NOTCH2 gRNA (GGCGCTCTGGCTGTGCTGCG) were designed using the Optimized CRISPR Design tool (F. Zheng, MIT) and cloned into the BsmBI site of plentiCRISPRv2. gRNA‐containing pLentiCRISPR plasmids were co‐transfected with pVSVG, pRSV‐REV, and pMDL packaging plasmids into HEK‐293T cells using calcium phosphate transfection. After 48 h, viral supernatants were collected, concentrated using PEG‐IT viral precipitator (SBI), and resuspended in PBS. Cells were transduced in growth medium overnight and selected with 2 µg mL^−1^ puromycin 48 h after infection. CRISPR modifications were verified by Western blot.

### Western Blotting

Cell lysates were prepared with equal amounts of total protein (as measured using the Pierce Coomassie protein assay reagent) and separated on a NuPage Bis‐Tris gels, transferred to PVDF (ThermoFisher), blocked in 5% milk and subjected to Western blot analysis using antibodies for Notch2 (Cell Signaling, D76A6) and GAPDH (Cell Signaling, D16H11). The blots were developed using ECL Western blot detection reagents (Pierce), and the signal was detected on iBright CL1500 Imaging System (ThermoFisher).

### Statistical Analysis

All statistical analysis was performed in GraphPad Prism 9.0 (GraphPad Software Inc.). Statistical significance and replicates are described in the figure legends. All graphical data were reported as means ± SEM. Significance levels were set at *P* < 0.033 (*), *P* < 0.002 (**), *P* < 0.001 (***).

## Conflict of Interest

The authors declare no conflict of interest.

## Supporting information

Supporting Information

Supplemental Movie 1

## Data Availability

That data generated in the course of this study are available from the corresponding author upon reasonable request.

## References

[1] H. G. Song , A. Lammers , S. Sundaram , L. Rubio , A. X. Chen , L. Li , J. Eyckmans , S. N. Bhatia , C. S. Chen , Adv. Funct. Mater. 2020, 30, 2003777. 33613149 10.1002/adfm.202003777 PMC7891457

[2] K. R. Stevens , M. D. Ungrin , R. E. Schwartz , S. Ng , B. Carvalho , K. S. Christine , R. R. Chaturvedi , C. Y. Li , P. W. Zandstra , C. S. Chen , S. N. Bhatia , Nat. Commun. 2013, 4, 1847. 23673632 10.1038/ncomms2853 PMC3660041

[3] K. R. Stevens , M. A. Scull , V. Ramanan , C. L. Fortin , R. R. Chaturvedi , K. A. Knouse , J. W. Xiao , C. Fung , T. Mirabella , A. X. Chen , M. G. McCue , M. T. Yang , H. E. Fleming , K. Chung , Y. P. de Jong , C. S. Chen , C. M. Rice , S. N. Bhatia , Sci. Transl. Med. 2017, 9, eaah5505. 28724577 10.1126/scitranslmed.aah5505 PMC5896001

[4] J. D. Baranski , R. R. Chaturvedi , K. R. Stevens , J. Eyckmans , B. Carvalho , R. D. Solorzano , M. T. Yang , J. S. Miller , S. N. Bhatia , C. S. Chen , Proc. Natl. Acad. Sci. USA 2013, 110, 7586. 23610423 10.1073/pnas.1217796110 PMC3651499

[5] B. Vroman , N. F. LaRusso , Lab. Invest. J. Tech. Methods Pathol. 1996, 74, 303. 8569194

[6] J. H. Tabibian , C. E. Trussoni , S. P. O'Hara , P. L. Splinter , J. K. Heimbach , N. F. LaRusso , Lab. Invest. 2014, 94, 1126. 25046437 10.1038/labinvest.2014.94 PMC4184949

[7] L. Loarca , T. M. D. Assuncao , N. Jalan‐Sakrikar , S. Bronk , A. Krishnan , B. Huang , L. Morton , C. Trussoni , L. M. Bonilla , E. Krueger , S. O'Hara , P. Splinter , G. Shi , M. J. L. Pisarello , G. J. Gores , R. C. Huebert , N. F. LaRusso , Lab. Invest. 2017, 97, 1385. 28892096 10.1038/labinvest.2017.63 PMC5664217

[8] M. Huch , C. Dorrell , S. F. Boj , J. H. van Es , V. S. W. Li , M. van de Wetering , T. Sato , K. Hamer , N. Sasaki , M. J. Finegold , A. Haft , R. G. Vries , M. Grompe , H. Clevers , Nature 2013, 494, 247. 23354049 10.1038/nature11826 PMC3634804

[9] M. Huch , H. Gehart , R. van Boxtel , K. Hamer , F. Blokzijl , M. M. A. Verstegen , E. Ellis , M. van Wenum , S. A. Fuchs , J. de Ligt , M. van de Wetering , N. Sasaki , S. J. Boers , H. Kemperman , J. de Jonge , J. N. M. Ijzermans , E. E. S. Nieuwenhuis , R. Hoekstra , S. Strom , R. R. G. Vries , L. J. W. van der Laan , E. Cuppen , H. Clevers , Cell 2015, 160, 299. 25533785 10.1016/j.cell.2014.11.050 PMC4313365

[10] F. Sampaziotis , M. C. de Brito , I. Geti , A. Bertero , N. R. Hannan , L. Vallier , Nat. Protoc. 2017, 12, 814. 28333915 10.1038/nprot.2017.011 PMC5467722

[11] O. C. Tysoe , A. W. Justin , T. Brevini , S. E. Chen , K. T. Mahbubani , A. K. Frank , H. Zedira , E. Melum , K. Saeb‐Parsy , A. E. Markaki , L. Vallier , F. Sampaziotis , Nat. Protoc. 2019, 14, 1884. 31110298 10.1038/s41596-019-0168-0

[12] F. Sampaziotis , A. W. Justin , O. C. Tysoe , S. Sawiak , E. M. Godfrey , S. S. Upponi , R. L. Gieseck , M. C. de Brito , N. L. Berntsen , M. J. Gómez‐Vázquez , D. Ortmann , L. Yiangou , A. Ross , J. Bargehr , A. Bertero , M. C. F. Zonneveld , M. T. Pedersen , M. Pawlowski , L. Valestrand , P. Madrigal , N. Georgakopoulos , N. Pirmadjid , G. M. Skeldon , J. Casey , W. Shu , P. M. Materek , K. E. Snijders , S. E. Brown , C. A. Rimland , I. Simonic , S. E. Davies , K. B. Jensen , M. Zilbauer , W. T. H. Gelson , G. J. Alexander , S. Sinha , N. R. F. Hannan , T. A. Wynn , T. H. Karlsen , E. Melum , A. E. Markaki , K. Saeb‐Parsy , L. Vallier , Nat. Med. 2017, 23, 954. 28671689 10.1038/nm.4360

[13] M. Ishii , B. Vroman , N. F. LaRusso , Gastroenterology 1989, 97, 1236. 2792660 10.1016/0016-5085(89)91695-8

[14] L. Tian , A. Deshmukh , Z. Ye , Y.‐Y. Jang , Stem Cell Rev. Rep. 2016, 12, 500. 27138846 10.1007/s12015-016-9657-5 PMC6186008

[15] J. A. McAteer , A. P. Evan , K. D. Gardner , Anat. Rec. 1987, 217, 229. 3578840 10.1002/ar.1092170303

[16] N. Tanimizu , A. Miyajima , K. E. Mostov , Mol. Biol. Cell 2007, 18, 1472. 17314404 10.1091/mbc.E06-09-0848 PMC1838984

[17] P. L. Lewis , J. Su , M. Yan , F. Meng , S. S. Glaser , G. D. Alpini , R. M. Green , B. Sosa‐Pineda , R. N. Shah , Sci. Rep. 2018, 8, 12220. 30111800 10.1038/s41598-018-30433-6 PMC6093899

[18] A. Funfak , L. Bouzhir , E. Gontran , N. Minier , P. Dupuis‐Williams , S. Gobaa , Front. Bioeng. Biotechnol. 2019, 7, 417. 31921820 10.3389/fbioe.2019.00417 PMC6923240

[19] Y. Du , G. Khandekar , J. Llewellyn , W. Polacheck , C. S. Chen , R. G. Wells , Hepatology 2020, 71, 1350. 31465556 10.1002/hep.30918 PMC7048662

[20] C. Chen , P. G. M. Jochems , L. Salz , K. Schneeberger , L. C. Penning , S. F. J. van de Graaf , U. Beuers , H. Clevers , N. Geijsen , R. Masereeuw , B. Spee , Biofabrication 2018, 10, 034103. 29848792 10.1088/1758-5090/aac8fd

[21] M. R. McGill , C. D. Williams , Y. Xie , A. Ramachandran , H. Jaeschke , Toxicol. Appl. Pharm. 2012, 264, 387. 10.1016/j.taap.2012.08.015 PMC3478469 22980195

[22] E. M. Blais , K. D. Rawls , B. V. Dougherty , Z. I. Li , G. L. Kolling , P. Ye , A. Wallqvist , J. A. Papin , Nat. Commun. 2017, 8, 14250. 28176778 10.1038/ncomms14250 PMC5309818

[23] M. Martignoni , G. M. M. Groothuis , R. de Kanter , Expert Opin. Drug Metab. Toxicol. 2006, 2, 875. 17125407 10.1517/17425255.2.6.875

[24] Y. Liu , C. Meyer , C. Xu , H. Weng , C. Hellerbrand , P. ten Dijke , S. Dooley , Am. J. Physiol. 2013, 304, G449. 10.1152/ajpgi.00199.2012 23275613

[25] D. T. Odom , R. D. Dowell , E. S. Jacobsen , W. Gordon , T. W. Danford , K. D. MacIsaac , P. A. Rolfe , C. M. Conboy , D. K. Gifford , E. Fraenkel , Nat. Genet. 2007, 39, 730. 17529977 10.1038/ng2047 PMC3797512

[26] S. R. Khetani , D. R. Berger , K. R. Ballinger , M. D. Davidson , C. Lin , B. R. Ware , J. Lab. Autom. 2014, 20, 216. 10.1177/2211068214566939 25617027

[27] B. Trappmann , B. M. Baker , W. J. Polacheck , C. K. Choi , J. A. Burdick , C. S. Chen , Nat. Commun. 2017, 8, 371. 28851858 10.1038/s41467-017-00418-6 PMC5575316

[28] D.‐H. T. Nguyen , E. Lee , S. Alimperti , R. J. Norgard , A. Wong , J. J.‐K. Lee , J. Eyckmans , B. Z. Stanger , C. S. Chen , Sci. Adv. 2019, 5, eaav6789. 31489365 10.1126/sciadv.aav6789 PMC6713506

[29] Y. Guan , D. Xu , P. M. Garfin , U. Ehmer , M. Hurwitz , G. enns , S. Michie , M. Wu , M. Zheng , T. Nishimura , J. Sage , G. Peltz , JCI Insight 2017, 2, e94954. 10.1172/jci.insight.94954 PMC5621886 28878125

[30] F. Sampaziotis , M. C. de Brito , P. Madrigal , A. Bertero , K. Saeb‐Parsy , F. A. C. Soares , E. Schrumpf , E. Melum , T. H. Karlsen , J. A. Bradley , W. T. H. Gelson , S. Davies , A. Baker , A. Kaser , G. J. Alexander , N. R. F. Hannan , L. Vallier , Nat. Biotechnol. 2015, 33, 845. 26167629 10.1038/nbt.3275 PMC4768345

[31] M. Ogawa , S. Ogawa , C. E. Bear , S. Ahmadi , S. Chin , B. Li , M. Grompe , G. Keller , B. M. Kamath , A. Ghanekar , Nat. Biotechnol. 2015, 33, 853. 26167630 10.1038/nbt.3294

[32] S. Perumal , O. Antipova , J. P. R. O. Orgel , Proc. Natl. Acad. Sci. USA 2008, 105, 2824. 18287018 10.1073/pnas.0710588105 PMC2268544

[33] T. Takebe , K. Sekine , M. Enomura , H. Koike , M. Kimura , T. Ogaeri , R.‐R. Zhang , Y. Ueno , Y.‐W. Zheng , N. Koike , S. Aoyama , Y. Adachi , H. Taniguchi , Nature 2013, 499, 481. 23823721 10.1038/nature12271

[34] G. Tian , C. D. Sobotka‐Briner , J. Zysk , X. Liu , C. Birr , M. A. Sylvester , P. D. Edwards , C. D. Scott , B. D. Greenberg , J. Biol. Chem. 2002, 277, 31499. 12072428 10.1074/jbc.M112328200

[35] S. Alimperti , T. Mirabella , V. Bajaj , W. Polacheck , D. M. Pirone , J. Duffield , J. Eyckmans , R. K. Assoian , C. S. Chen , Proc. Natl. Acad. Sci. USA 2017, 114, 8758. 28765370 10.1073/pnas.1618333114 PMC5565405

[36] S. N. Bhatia , G. H. Underhill , K. S. Zaret , I. J. Fox , Sci. Transl. Med. 2014, 6, 245sr2. 25031271 10.1126/scitranslmed.3005975 PMC4374645

[37] K.‐J. Jang , M. A. Otieno , J. Ronxhi , H.‐K. Lim , L. Ewart , K. R. Kodella , D. B. Petropolis , G. Kulkarni , J. E. Rubins , D. Conegliano , J. Nawroth , D. Simic , W. Lam , M. Singer , E. Barale , B. Singh , M. Sonee , A. J. Streeter , C. Manthey , B. Jones , A. Srivastava , L. C. Andersson , D. Williams , H. Park , R. Barrile , J. Sliz , A. Herland , S. Haney , K. Karalis , D. E. Ingber , G. A. Hamilton , Sci. Transl. Med. 2019, 11, eaax5516. 31694927 10.1126/scitranslmed.aax5516

[38] X. Chen , Y. S. Zhang , X. Zhang , C. Liu , Bioact. Mater. 2021, 6, 1012. 33102943 10.1016/j.bioactmat.2020.09.022 PMC7566214

[39] Y. Zong , A. Panikkar , J. Xu , A. Antoniou , P. Raynaud , F. Lemaigre , B. Z. Stanger , Development 2009, 136, 1727. 19369401 10.1242/dev.029140 PMC2673761

[40] J. J. Hofmann , A. C. Zovein , H. Koh , F. Radtke , G. Weinmaster , M. L. Iruela‐Arispe , Development 2010, 137, 4061. 21062863 10.1242/dev.052118 PMC2976287

[41] M. Kitade , V. M. Factor , J. B. Andersen , A. Tomokuni , K. Kaji , H. Akita , A. Holczbauer , D. Seo , J. U. Marquardt , E. A. Conner , S.‐B. Lee , Y.‐H. Lee , S. S. Thorgeirsson , Gene Dev. 2013, 27, 1706. 23913923 10.1101/gad.214601.113 PMC3744728

[42] G. K. Michalopoulos , W. C. Bowen , K. Mulè , J. Luo , Gene Expression 2003, 11, 55. 12837037 10.3727/000000003108748964 PMC1913286

[43] A. Martinez‐Hernandez , P. S. Amenta , Virchows Archiv A Pathological Anatomy and Histopathology 1993, 423, 77. 8212543 10.1007/BF01606580

[44] N. Shiojiri , Y. Sugiyama , Hepatology 2004, 40, 346. 15368439 10.1002/hep.20303

[45] A. Totaro , M. Castellan , G. Battilana , F. Zanconato , L. Azzolin , S. Giulitti , M. Cordenonsi , S. Piccolo , Nat. Commun. 2017, 8, 15206. 28513598 10.1038/ncomms15206 PMC5442321

[46] K. B. Kaylan , I. C. Berg , M. J. Biehl , A. Brougham‐Cook , I. Jain , S. M. Jamil , L. H. Sargeant , N. J. Cornell , L. T. Raetzman , G. H. Underhill , Elife 2018, 7, e38536. 30589410 10.7554/eLife.38536 PMC6342520

[47] C. A. Rimland , S. G. Tilson , C. M. Morell , R. A. Tomaz , W.‐Y. Lu , S. E. Adams , N. Georgakopoulos , F. Otaizo‐Carrasquero , T. G. Myers , J. R. Ferdinand , R. L. Gieseck , F. Sampaziotis , O. C. Tysoe , A. Ross , J. M. Kraiczy , B. Wesley , D. Muraro , M. Zilbauer , G. C. Oniscu , N. R. F. Hannan , S. J. Forbes , K. Saeb‐Parsy , T. A. Wynn , L. Vallier , Hepatology 2021, 73, 247. 32222998 10.1002/hep.31252 PMC8641381

[48] G. Mehta , C. M. Williams , L. Alvarez , M. Lesniewski , R. D. Kamm , L. G. Griffith , Biomaterials 2010, 31, 4657. 20304480 10.1016/j.biomaterials.2010.01.138 PMC2872479

[49] J. G. Rheinwatd , H. Green , Cell 1975, 6, 331. 1052771 10.1016/s0092-8674(75)80001-8

