# Supplementary material for: Directing Cholangiocyte Morphogenesis in Natural Biomaterial Scaffolds
Source: Adv Sci (Weinh). 2021 Nov 16;9(3):2102698. doi: 10.1002/advs.202102698 (PMC8787431; doi:10.1002/advs.202102698)
Supplement: Supplementary file 1 — Supporting Information [file ADVS-9-2102698-s001.pdf]

ADVANCED SCIENCE
Open Access

Supporting Information

for *Adv. Sci.*, DOI: 10.1002/advs.202102698

Directing Cholangiocyte Morphogenesis in Natural Biomaterial Scaffolds

Quinton Smith, Jennifer Bays, Linqing Li, Haniyah Shareef, Christopher Chen, Sangeeta Bhatia

Supplementary Figures

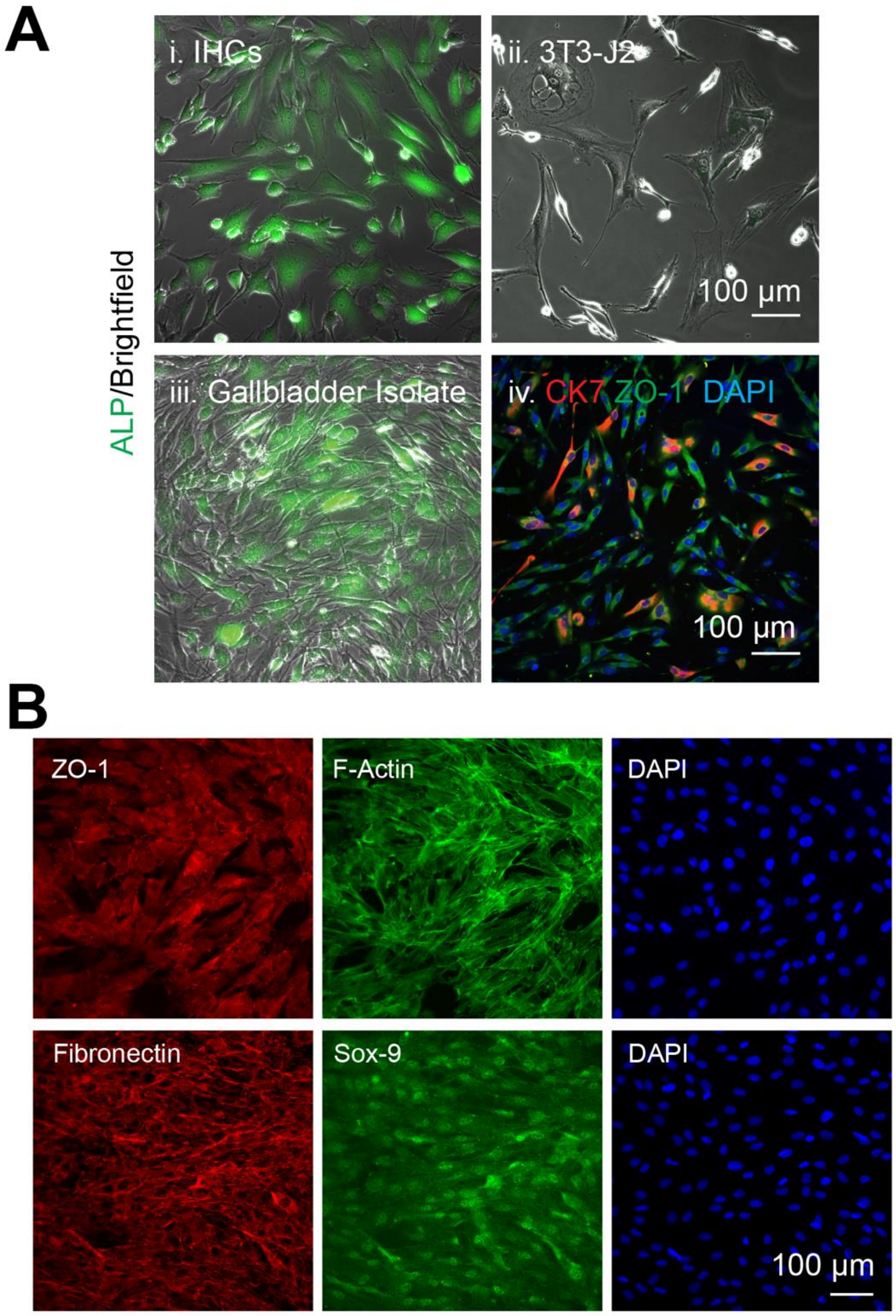

Supplementary Figure 1. Characterization of Cholangiocyte Populations. (A) Alkaline phosphatase (ALP) uptake comparison between (i) intrahepatic cholangiocytes (IHCs), (ii) control J2-3T3 mouse fibroblasts and (iii) fresh extrahepatic cholangiocyte (EHCs) isolates from human gallbladder tissue. (iv) Immunofluorescence staining of fresh EHCs.

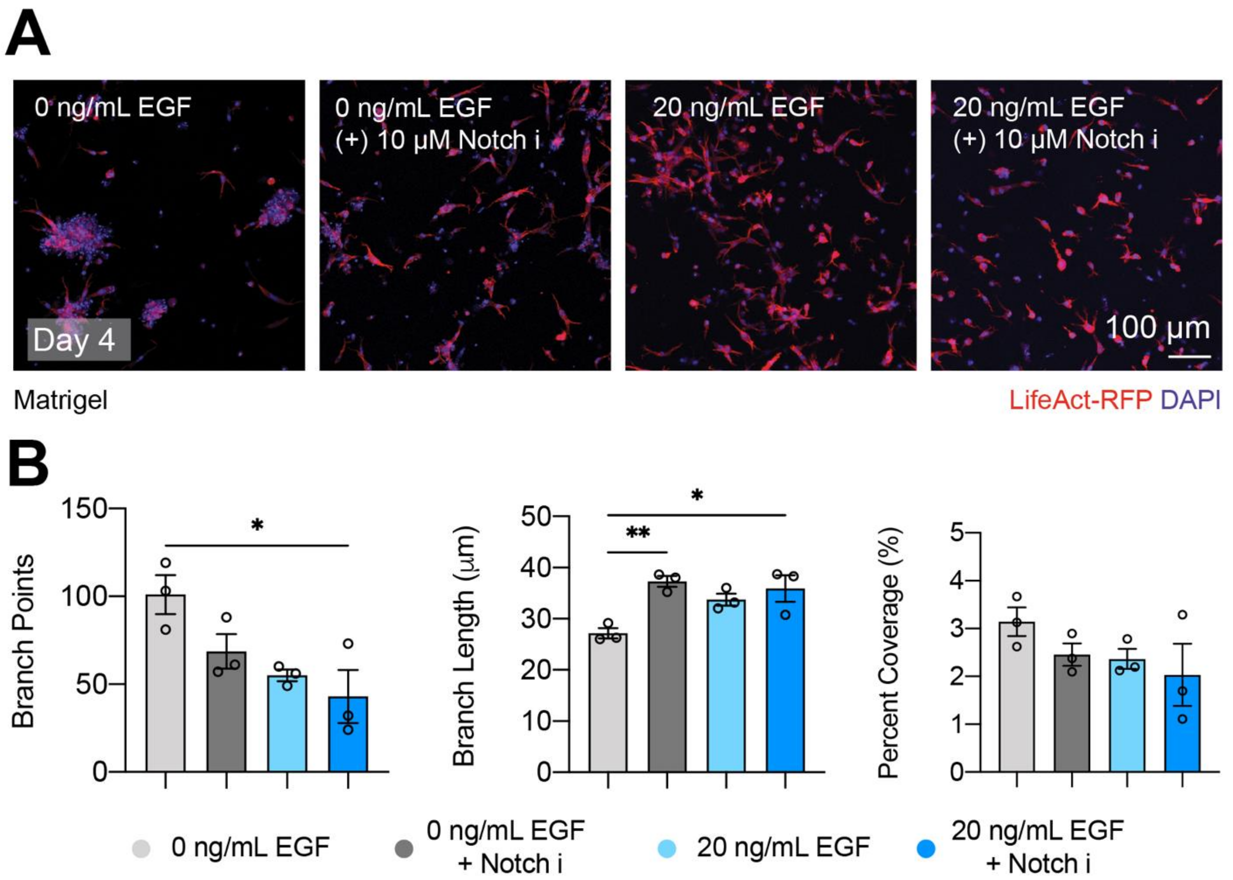

Supplementary Figure 2. 3D Culture in Matrigel Does Not Support Cholangiocyte Branching Morphogenesis. (A) Representative images of LifeAct RFP-IHCs encapsulated in Matrigel, cultured with and without EGF or Notch inhibition (10 µM L,685,458). **(B)** Quantification of branch length, points, and network percent coverage. Image data were generated from at least 3 independent fields of view from 3 biological replicate experiments. P-values were obtained by a One-Way ANOVA Tukey's hypothesis test. $P < 0.033$ (*), $P< 0.002$ (**), $P <0.001$ (***). All data represented as mean ± SEM.

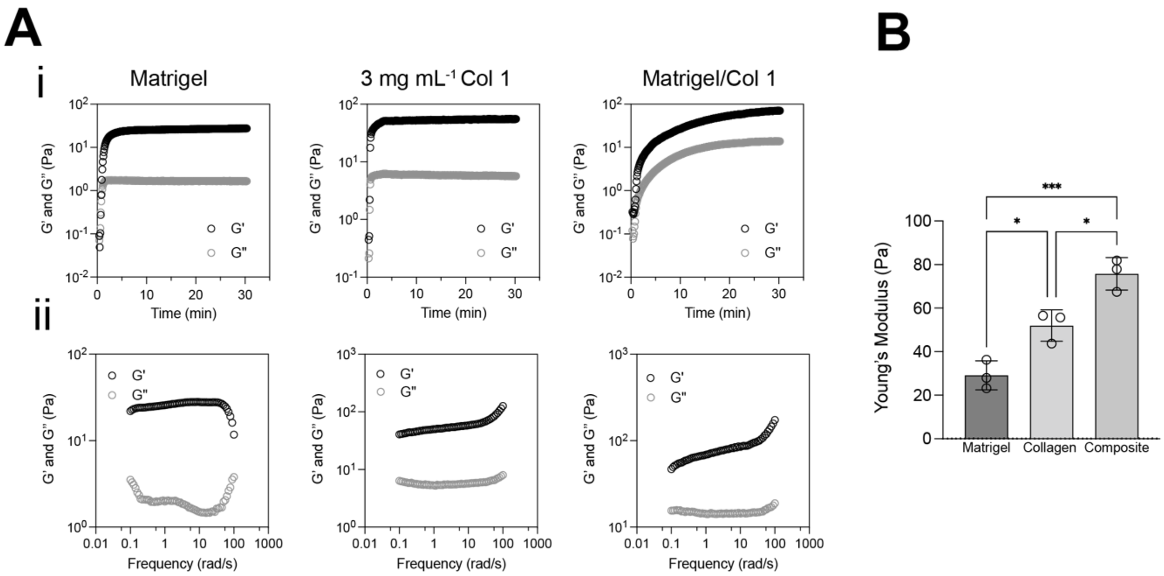

Supplementary Figure 3. Rheological characterization of collagen type 1, Matrigel and composite gels. (A, i) Dynamic time sweeps of the viscoelastic modulus (G' storage and G" loss modulus) of Matrigel, 3 mg mL^{-1} collagen 1 and 50% (v/v) Matrigel/3 mg mL^{-1} collagen composite gels**. (A, ii)** Dynamic frequency sweeps of G' and G'' across the various materials. **(B)** Empirically derived Young's Moduli of Matrigel, collagen type 1 and composite hydrogels (n = 3 independent gels).

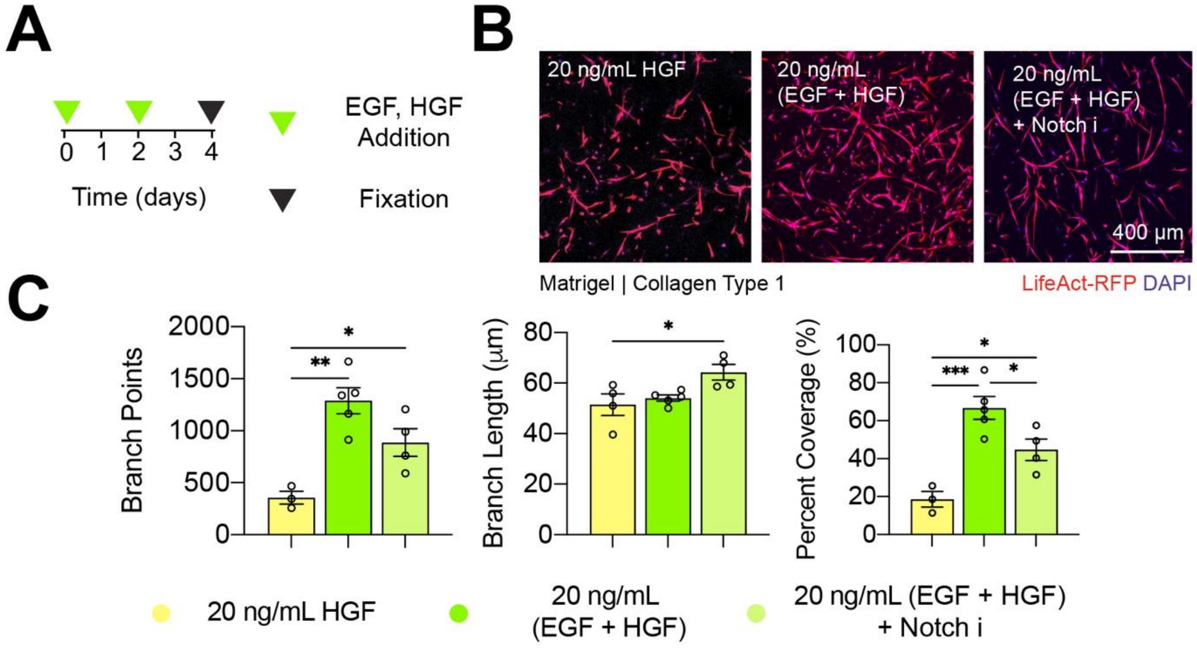

Supplementary Figure 4. Hepatocyte Growth Factor (HGF) Supplementation Enhances Cholangiocyte Branching in Matrigel/Collagen Type 1 Hydrogel Blends. (A) Experimental approach to test the roles of HGF supplementation on IHC-RFP branching. (B) Representative maximum intensity projection of z-stack confocal images taken four days post encapsulation. (C) Quantification of percent coverage, major and minor axis length from at least 3 different fields of view from biological replicate experiments (n =3). P-values were obtained by a One-Way ANOVA Tukey's hypothesis test. $P < 0.033$ (*), $P < 0.002$ (**), $P < 0.001$ (***). All data represented as mean ± SEM.

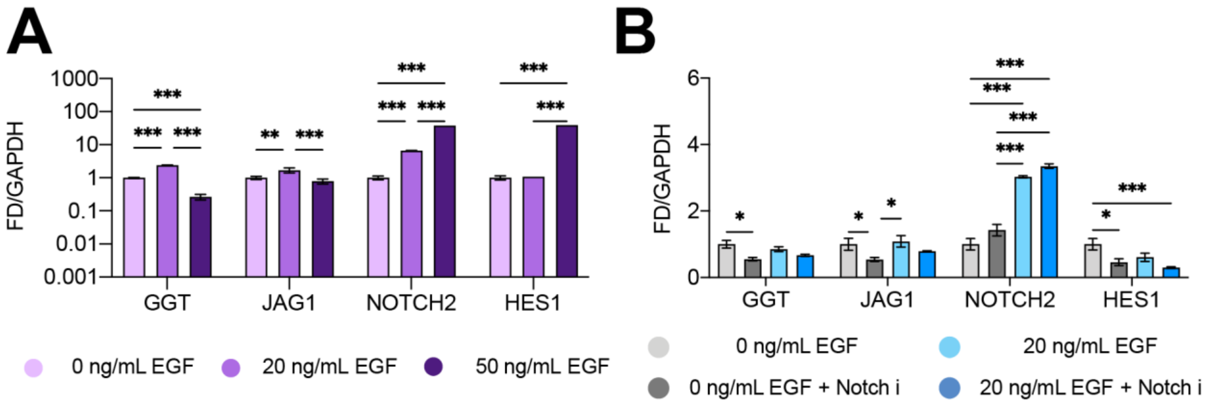

Supplementary Figure 5. Effects of EGF Stimulation on Notch Signaling in 2D. (A) mRNA expression of GGT, JAG1, NOTCH2 and HES1 of IHCs cultured in the same dose regiment as 3D cultures (media exchanged every two days) with 0, 20 and 50 ng mL^{-1} EGF stimulation. Data is shown from pooled independent 2D cultures (n = 3 biological replicates). (B) mRNA expression of the aforementioned genes, of IHCs cultured in 2D, under 0 and 20 ng mL^{-1} EGF stimulation with and without Notch inhibition. Cells were harvested after 4 days following media exchanges every two days. P-values were obtained via Two-Way ANOVA Tukey's hypothesis testing. P < 0.033 (*), P< 0.002 (**), P <0.001 (***). All data represented as mean ± SEM.

Supplemental Movie 1. Confocal image of NRCs Grow in Microfluidic Channel.

Supplementary Table 1: Primary and Secondary Antibodies

Supplementary Table 2: Reagents

Supplementary Table 3: Primer Set

Table S1: Antibodies

Primary Antibodies	Source	Cat no	Host Species	Dilution
Cytokeratin-19	Abcam	ab7754	Mouse	1:200
Cytokeratin-7	Abcam	ab181598	Rabbit	1:100
CFTR	Abcam	ab2784	Mouse	1:200
Fibronectin	Abcam	ab2413	Rabbit	1:250
ZO-1	Invitrogen	40-2200	Rabbit	1:100
β-catenin	Cell Signaling	L54E2 (2677S)	Mouse	1:5
HNF-1β	Santa Cruz Biotechnology	sc-130407	Mouse	1:200
SOX9	Santa Cruz Biotechnology	sc-166505	Mouse	1:200
Alexa Fluor 546	Life Technologies (Eugene, OR)	A11010	Goat anti-rabbit	1:500
Alexa Fluor 546	Life Technologies	A11003	Goat anti-mouse	1:500
Alexa Fluor 488	Life Technologies	A21206	Donkey anti-rabbit	1:500
Alexa Fluor 647	Life Technologies	A22287	Phalloidin	1:1000
Cytokeratin 7 Antibody (RCK105) PE	Santa Cruz Biotechnology	sc-23876 PE	Human	1:200
AFP Antibody (C3) Alexa Fluor® 647	Santa Cruz Biotechnology	sc-8399 AF647	Human	1:200
Anti-alpha 1 Antitrypsin antibody [EPR9090] (Alexa Fluor® 647)	Abcam	ab206735	Human	1:200
PE Mouse Anti-Human IgG	BD Biosciences	555787	Human	1:200

Alexa Fluor® 647 Mouse IgG1 κ Isotype Control	BD Biosciences	557714		1:200
DAPI	ThermoFisher	D1306		1:50,000

Table S2: Reagents

Chemicals, Peptides and Recombinant Proteins	Source	Cat No.
Alkaline Phosphatase Live Stain	Life Technologies	A14353
Rhodamine 123 (100 µM)	Sigma-Aldrich	83702-10MG
γ-Glutamyl Transferase (GGT) Activity Fluorometric Assay Kit	Sigma-Aldrich	MAK090-1KT
Recombinant Human EGF	R&D	236-EG-200
L-685,458 (Notch Inhibitor)	Tocris Biosciences	2627
rLV-Ubi-LifeAct TagRFP Lentiviral Vectors	Ibidi	60142

Table S3: Primer Set

Primers	Source	Assay ID
GGT	Life Technologies	Hs00980756_m1 FAM
JAGGED1	Life Technologies	Hs01070032_m1 FAM
NOTCH2	Life Technologies	Hs01050702_m1 FAM
HES1	Life Technologies	Hs00172878_m1 FAM
